# Characterization of a mobilizable megaplasmid carrying multiple resistance genes from a clinical isolate of *Pseudomonas aeruginosa*

**DOI:** 10.3389/fmicb.2023.1293443

**Published:** 2023-11-27

**Authors:** Li Mei, Yang Song, Dongxin Liu, Yixiao Li, Li Liu, Keyi Yu, Mengnan Jiang, Duochun Wang, Qiang Wei

**Affiliations:** ^1^National Pathogen Resource Center, Chinese Center for Disease Control and Prevention, Beijing, China; ^2^Division of Infectious Disease, Key Laboratory of Surveillance and Early-Warning on Infectious Disease, Chinese Center for Disease Control and Prevention, Beijing, China; ^3^National Institute for Communicable Disease Control and Prevention, Chinese Center for Disease Control and Prevention, Beijing, China

**Keywords:** *Pseudomonas aeruginosa*, mobilizable plasmid, conjugation transfer, *tmexCD-oprJ*, *bla*_DIM−1_, *qnrVC6*, *mer* genes

## Abstract

**Introduction:**

The horizontal transfer of antibiotic resistance genes mediated by plasmids seriously hinders the effectiveness of modern medical treatment, and thus has attracted widespread attention. Additionally, the co-selection mechanism of antibiotic resistance genes (ARGs) and heavy metal resistance genes (MRGs) on mobile elements may further exacerbate the horizontal transfer of resistance genes.

**Methods:**

In this study, a multidrug-resistant *Pseudomonas aeruginosa* strain, termed BJ86 (CHPC/NPRC1.4142), was isolated from a patient's sputum specimen. *In vitro* tests for antimicrobial susceptibility, conjugation, whole-genome sequencing, and bioinformatics analysis were used to explore the potential mechanisms of resistance and its spread.

**Results and discussion:**

Sequencing analysis indicates that *P. aeruginosa* BJ86 carries an amazing 522.5 kb-length megaplasmid, pBJ86, which contained a 93.5 kb-length multiple resistance region (MRR); 18 kinds of genes were identified as ARGs in this region, including *tmexCD-oprJ*, *bla*_DIM−1_, *qnrVC6* that mediate resistance to multiple antibiotics and the operons *mer* that mediates heavy metal mercury resistance. In addition, there is also an 80 kb variable region (VR) on the plasmid pBJ86, and the genes encoding relaxase and type IV coupling protein (T4CP) were determined in this region, both of which are related to the conjugation and transfer ability of the plasmid. Bioinformatics analysis shows that many functional genes have insertion sequences and transposases on their flanks, which may have accumulated in the plasmid pBJ86 after multiple acquisition events. Conjugated transfer and *in vitro* tests for antimicrobial susceptibility verified the mobility and plasmid pBJ86-mediated resistance. To our knowledge, we are the first to report a mobilizable megaplasmid that simultaneously carried *tmexCD-oprJ*, *bla*_DIM−1_, *qnrVC6*, and the operons *mer* and can be transferred with frequencies of 6.24 × 10^−7^ transconjugants per donor cell.

## 1 Introduction

*Pseudomonas aeruginosa* is a Gram-negative opportunistic pathogen that is one of the most common causes of acute infection in patients with immune dysfunction or other susceptible diseases, often leading to poor prognosis in critically ill patients (Sievert et al., 2013; Nathwani et al., 2014; Bassetti et al., 2018a,b). In the United States, *P. aeruginosa* has been listed by the Infectious Diseases Society of America as one of the six most dangerous clinical pathogens worldwide. In China, *P. aeruginosa* has also been one of the most common Gram-negative bacteria in clinical practice in the past 20 years, with a prevalence rate third only to *Klebsiella pneumoniae* and *Escherichia coli* (Hu et al., 2016).

Due to the inherent structure and characteristics of *P. aeruginosa*, it has a high intrinsic resistance to many antibiotics (Pang et al., 2019). Meanwhile, bacteria can also obtain antibiotic resistance genes (ARGs) through spontaneous genetic mutations or horizontal gene transfer (HGT). The former can mainly be transmitted to offspring through vertical gene transfer (VGT), while the latter has the potential to spread ARGs faster and more extensively, significantly promoting the emergence and development of multidrug-resistant strains (Li and Zhang, 2022). For *P. aeruginosa*, *tmexCD*−*oprJ, bla*_DIM−1_*, and qnrVC6*, can encode resistance to different types of antibiotics. The *tmexCD-oprJ* Operon encodes an efflux pump, and its expression is usually affected by the regulatory factor *nfxB*. When it is overexpressed, it is usually related to the resistance of levofloxacin, ciprofloxacin, and other fluoroquinolones, but it can also pump out other antibiotics, such as macrolide and tetracyclines. In addition, mutations in *mexD* may also alter the substrate specificity of efflux pumps, which is related to changes in bacterial resistance to antibiotics such as carbicillin and ceflozan-tazobactam (Gomis-Font et al., 2021; Lorusso et al., 2022). As a class B metallo-β-lactamase (MBL) gene, *bla*_DIM_ mainly exists on plasmids and mediates resistance to almost all β-lactams, including broad-spectrum cephalosporin and carbapenem, but does not affect monobactams (Tada et al., 2017). *qnrVC6* is a newly emerging quinolone resistance gene in *Pseudomonas* sp., which may have an additive effect of quinolone resistance together with other genes in the *qnr* family, thus helping to obtain full quinolone resistance (Liu et al., 2018). Therefore, the coexistence of *mexCD-oprJ* with *bla*_DIM_, *qnrVC6*, and other ARGs in mobile elements can accelerate the spread of ARGs and pose a serious threat to the effectiveness of current clinical treatment.

Recent research has shown that many substances, including antibiotics, non-antibiotic drugs, and environmental pollutants, can promote HGT (Alav and Buckner, 2023). In addition, the selective pressure exerted by heavy metals on pathogenic microorganisms is increasingly being recognized as a critical driving factor in promoting the selection and spread of antibiotic resistance in human and animal food chains (Capita and Alonso-Calleja, 2013). Heavy metals with sublethal concentrations can induce bacterial resistance (Di Cesare et al., 2016) and promote the horizontal transfer of resistance genes (Wang et al., 2015), a direct or indirect factor affecting antibiotic resistance. Heavy metal resistance genes (MRGs) have been found to often coexist with ARGs on plasmids, and it allows bacteria to obtain MRGs while also obtaining ARGs through co-selection mechanisms and vice versa (Baker-Austin et al., 2006). This potential mechanism may further exacerbate the horizontal transfer of ARGs.

This study describes the gene structure and characteristics of a mobilizable multiple resistance megaplasmid pBJ86 carried from a clinical isolate of *P. aeruginosa*, which can transfer via conjugation. The plasmid simultaneously carries ARGs *tmexCD*−*oprJ, bla*_DIM−1_*, qnrVC6*, and MRGs in the operon *mer*.

## 2 Materials and methods

### 2.1 Bacterial strain and identification

In 2019, *P. aeruginosa* strain BJ86 (CHPC/NPRC1.4142) was isolated from a patient's sputum at the Friendship Hospital in Beijing, China. The strain is stored at the National Pathogen Resource Center (NPRC). Bacterial identification was performed using matrix-assisted laser desorption ionization time-of-flight mass spectrometry (MALDI-TOF MS).

### 2.2 Susceptibility testing

Minimum inhibitory concentrations (MICs) of 14 antimicrobial agents (Aztreonam, Cefepime, Ceftazidime, Piperacillin-zazobactam, Imipenem, Meropenem, Ciprofloxacin, Levofloxacin, Norfloxacin, Amikacin, Gentamicin, Tobramycin, Fosfomycin w/G6P, and Colistin) were detected by BD Phoenix™ M50 with NMIC-413. The results of Fosfomycin w/G6P refer to EUCAST (The European Committee on Antimicrobial Susceptibility Testing, 2020) and the results of other antibiotics were interpreted following the CLSI guidelines (Clinical Laboratory Standards Institute, 2023). *P. aeruginosa* ATCC27853 and *Escherichia coli* ATCC 25922 were used as control strains.

### 2.3 Genome sequencing and assembly

The genomes of the strains involved in the study were extracted by the Wizard Genomic DNA Extraction Kit (Promega, Madison, WI, USA) following the manufacturer's instructions and sequenced using the Illumina NovaSeq6000 and Oxford Nanopore Technologies MinION platforms. In order to improve the reliability of subsequent information analysis results, Sickle (https://githubcom/najoshi/sickle) was first used to process the raw data to obtain clean data. After sample quality control, clean data was assembled using Unicycler (https://github.com/rrwick/Unicycler) with the hybrid assembly strategy (Wick et al., 2017).

### 2.4 Bioinformatics analysis

Based on whole genome sequencing (WGS) results, further verification of the bacterial strain using EzBioCloud databases (https://www.ezbiocloud.net/tools/ani) was carried out to calculate Average Nucleotide Identity (ANI). The MLST2.0 databases was used to perform Multilocus sequence typing (MLST), the Resfinder4.1 databases (https://cge.food.dtu.dk/services/ResFinder/) to search for resistance genes, and the PlasmidFinder database (https://cge.food.dtu.dk/services/PlasmidFinder/) to identify plasmind type. All three databases are located in the Center for Genomic Epidemiology (CGE) server. The TAfinder database (https://bioinfo-mml.sjtu.edu.cn/TAfinder/index.php) was used to predict the Type II toxin-antitoxin (TA) systems, the tRNAscan SE 1.3.1 databases (http://lowelab.ucsc.edu/tRNAscan-SE/) to predict tRNA, codon 1.4.4-4 to analyze codon bias, the CRISPRCasFinder database (https://crisprcas.i2bc.paris-saclay.fr/) to predict CRISPR, and the PHASTER database (http://phaster.ca) to carry out annotation phase sequences. Prokka1.14.6 was used for annotation, using default parameters. BLAST was utilized in the NCBI database to search for sequences with higher plasmid coverage and consistency in GenBank compared to this study. Sequence comparisons and map generation were performed using BRIG and Easyfig (Sullivan et al., 2011).

### 2.5 Plasmid conjugation method

The conjugation assay was performed by using BJ86 as the donor and rifampicin-resistant *P. aeruginosa* PAO1 as the recipient to identify the self-transfer ability of the plasmid in this study. Rifampicin (32 μg/mL) and meropenem (8 μg/mL) were the antibiotics and concentrations used for selection. The donor and recipient bacteria were mixed in a 1:1 volume ratio and shaken at 37°C and 180 r/min for 10 h. Next, 200 μL of the mixed liquid after cultivation was taken and dropped onto a dual-resistance plate [the Brain-Heart Infusion (BHI) agar plates containing 32 μg/mL rifampicin and 8 μg/mL meropenem]. After this, the mixed liquid was inverted and cultured at 37°C for 10–24 h. Successful conjugation was confirmed by detecting ARGs, susceptibility testing, and MLST of suspected transconjugants. The calculation formula for conjugation frequency is as follows: conjugation frequency = number of transconjugant bacterial colonies × 10^*x*^/number of donor bacterial colonies × 10^*y*^ (*x, y* is the dilution factor). The growth kinetics of stains were detected using Bioscreen fully automated microbial growth curve analyzer under conditions of 37°C and OD_600_.

### 2.6 Nucleotide sequence accession number

The complete sequences of the plasmid pBJ86 and the chromosomes of BJ86 were submitted to GenBank under accession numbers CP133755 and CP133756, respectively. In addition, the complete genome sequence of strain BJ86 can be obtained through the National Microbiology Data Center (www.nmdc.cn) using the number NMDC60134482.

## 3 Results

### 3.1 General features of the stain BJ86 and plasmid pBJ86 sequence

**Figure 1:**
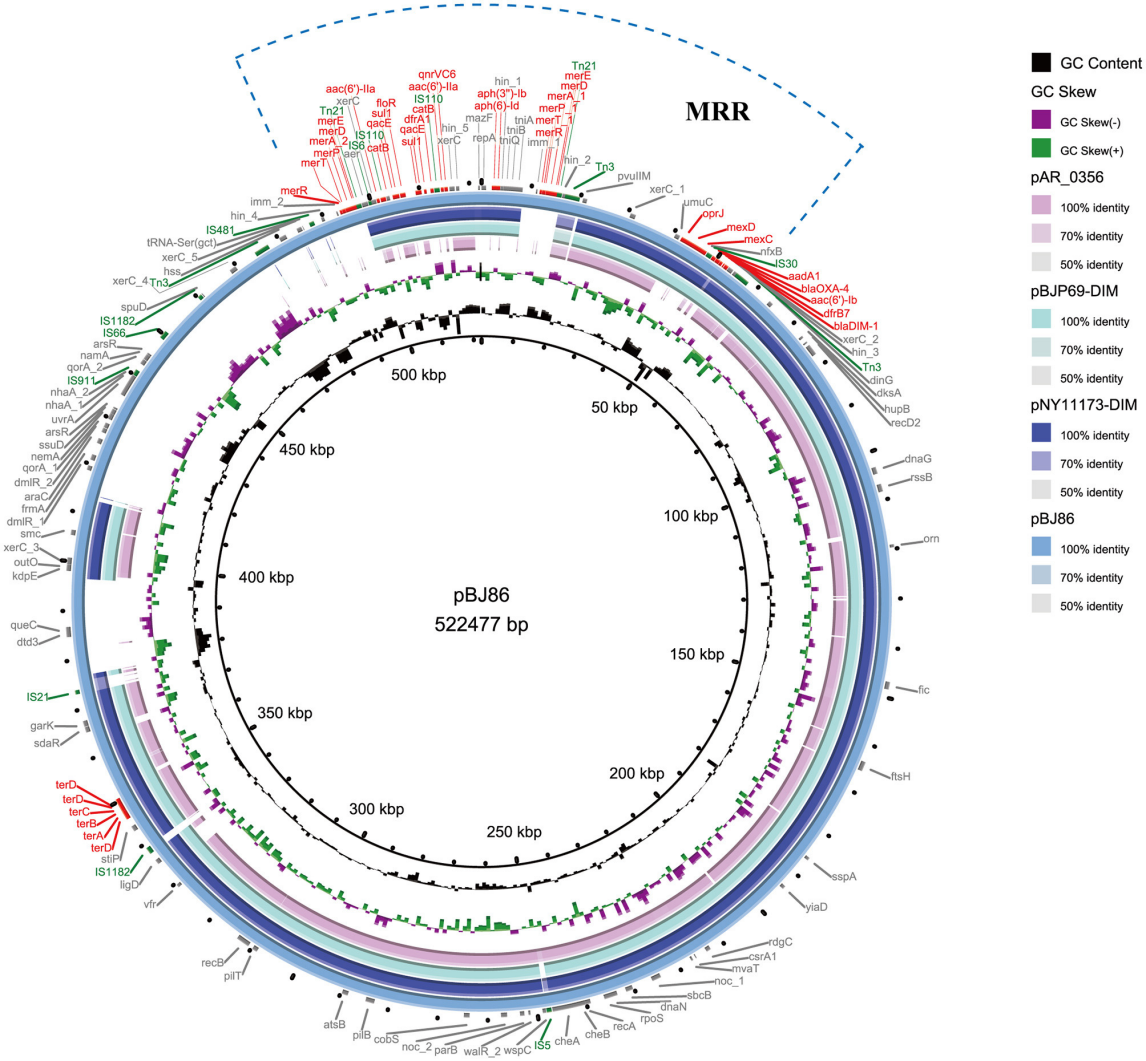
Comparative structural analysis of pBJ86 with other similar plasmids available in the NCBI nr database. The outermost circle represents the reference plasmid pBJ86. Genes involved in antimicrobial and heavy metal resistance are red, mobile elements are green, and genes involved in other functions are gray.

Genome *P. aeruginosa* BJ86, including a complete circular chromosome sequence (6,865,712 bp, GC accounting for 65.89%, MLST sequence type, ST2446 type) and a circular plasmid (pBJ86) sequence (522,477 bp, GC accounting for 56.62%), were identified. The plasmid pBJ86 has 599 predicted coding sequences (CDS), and 80.1% (480/599) of the CDS encode proteins of undetermined function (Figure 1; Supplementary Table S1). Comparing the complete sequence of pBJ86 with the plasmid sequence in Genbank, it was found that this sequence has the highest similarity to the plasmid pNY11173-DIM (Sequence ID: CP096957.1) from *P. aeruginosa* strain NY11173 (99.84% identity at 77% coverage), plasmid pBJP69-DIM (Sequence ID: MN208064.1) from *Pseudomonas sp*. strain BJP69 (99.87% identity at 77% coverage), and plasmid unnamed2 (Sequence ID: CP027170.1) from *P. aeruginosa* strain AR_0356 (98.07% identity at 68% coverage) (Figure 1).

### 3.2 Susceptibility testing of BJ86 and resistance genes

*P. aeruginosa* BJ86 is resistant to five categories and 10 agents of antibiotics, including two aminoglycosides (gentamicin and tobramycin), two antipseudomonal carbapenems (imipenem and meropenem), two antipseudomonal cephalosporins (cefepime and ceftazidime), three antipseudomonal fluoroquinolones (ciprofloxacin, levofloxacin, and norfloxacins), and one antipseudomonal penicillin + β-lactamase inhibitors (piperacillin-tazobactam) (Table 1). Due to the strain BJ86 being non-susceptible to at least one agent in ≥3 antimicrobial categories listed in Table 1, it belongs to a multidrug-resistant (MDR) strain (Magiorakos et al., 2012).

**Table 1:** Antimicrobial resistance pattern and resistant genes identified in *Pseudomonas aeruginosa* strain BJ86.

**Antimicrobial class/agent**	**MIC(μg/mL)**	**Resistance genes**	
	**(Susceptibility)**	**Plasmid**	**Chromosome**
**Aminoglycosides**			
Amikacin	8[S]		
Gentamicin	>8[R]	*aac(6′)-IIa*	
Tobramycin	>8[R]	*aac(6′)-Ib-Hangzhou, aac(6′)-IIa*	
**Antipseudomonal carbapenems**			
Imipenem	>8[R]	*bla*_DIM−1_	
Meropenem	>8[R]	*bla*_DIM−1_	
**Antipseudomonal cephalosporins**			
Cefepime	>16[R]	*bla*_OXA − 4_	*bla*_PAO_
Ceftazidime	>32[R]	*bla*_DIM−1_	*bla*_PAO_
**Antipseudomonal fluoroquinolones**			
Ciprofloxacin	>4[R]	*qnrVC6*	*crpP*
Levofloxacin	>8[R]	*tmexCD-OprJ*	
Norfloxacins	>8[R]	*tmexCD-OprJ*	
**Antipseudomonal penicillins** + β**-lactamase inhibitors**			
Piperacillin-Tazobactam	>64/4[R]	*bla*_DIM−1_	
**Monobactams**			
Aztreonam	8[S]		
**Phosphonic acids**			
Fosfomycin w/G6P	64[ < ECOFF]		*fosA*
**Polymyxins**			
Colistin	≤ 1[S]		

R, resistance; I, intermediate; S, susceptible; ECOFF, epidemiological break point based on *in vitro* drug sensitivity data.

There are six kinds of ARGs [$aph\left(3^{\prime }\right)-IIb,fosA,crpP,bla_{\text{PAO}}$, *bla*_OXA − 50_, and *catB7*] carried on the chromosome. The plasmid carries 16 kinds of ARGs, including six aminoglycoside resistance genes [*aph (3*^′′^*)-Ib, aph (6*^′′^*)-Id, aac (6*′*)-Ib cr, aadA1, aac (6*′*)-Ib Hangzhou, aac (6*′*)* and*- IIa*], two β-lactamase-encoding genes (*bla*_DIM−1_ and *bla*_OXA − 4_), one quinolone resistance gene (*qnrVC6*), one sulfonamide resistance gene (*sul1*), two trimethoprim resistance genes (*dfrA1 two dfrB7*), one quaternary ammonium compound resistance gene (*qacE*), one flufenicol resistance gene (*floR*), one chloramphenicol resistance genes (*catB*), and one operon *tmexCD-OprJ* encoding the antibiotic efflux pump. There are also two kinds of MRGs, including two structurally similar operons to *mer* that mediate resistance to mercury and one operon, *ter*, that mediates resistance to the oxygen ion form of tellurium.

### 3.3 Genetic contexts of multiple resistance region

**Figure 2:**
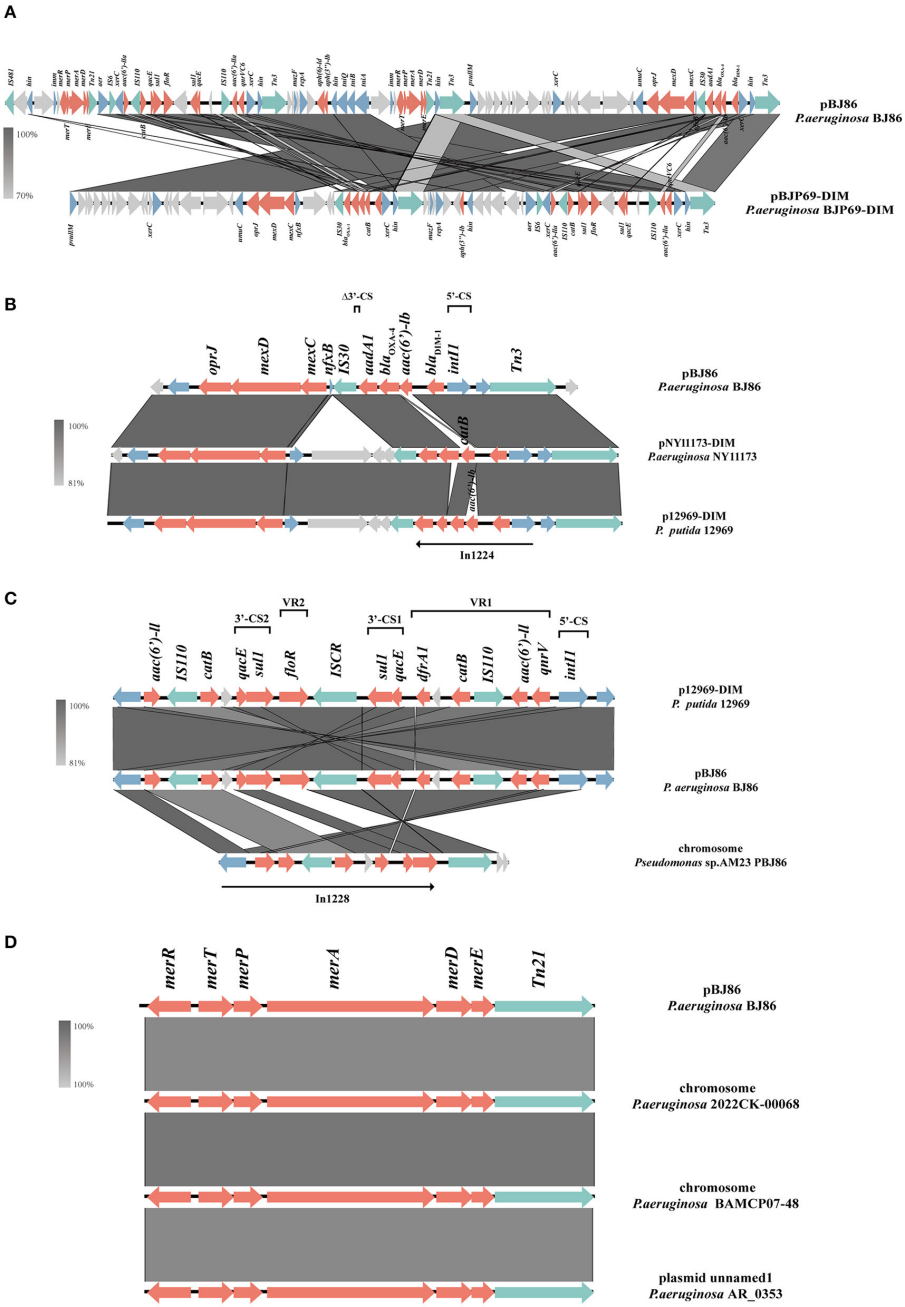
Schematic presentation of multiple resistance regions. Genes are presented as arrows, with the arrowhead indicating the direction of transcription. Genes involved in antimicrobial resistance are in red, mobile elements are in green, genes involved in other functions are in blue, and genes involved in undetermined coding functions are in light gray. Homologous segments generated by a BLASTn comparison (>70% identity) are gray boxes. Alignment of genetic contexts of the MRR with a length of 93.5 kb **(A)**, *tmexCD-oprJ* **(B)**, *bla*_DIM−1_ **(B)**, *qnrVC6* **(C)**, and *merRTPADE* **(D)** in the plasmid pBJ86-MRR are with similar sequences.

There is a multiple resistance region (MRR) with a length of 93.5 kb on plasmid pPBJ86, which contains 107 CDS and a GC content of 58%. In the MRR, a total of 18 resistance genes were detected (Figure 2A). Among them, the operon *tmexCD-oprJ* encoding an efflux pump and the ARGs *bla*_*DIM*−1_*, qnrVC6* can mediate the resistance of *P. aeruginosa* to multiple antibiotics, and the *mer* genes mediate the resistance to mercury.

Compared to pNY11173-DIM (Sequence ID: CP096957.1) and p12969-DIM (Sequence ID: KU130294.1), pBJ86 also has a complete genome structure encoding efflux pump and its regulatory factors *nfxB*, but it is evident that pBJ86 has a truncated *nfxB* (Figure 2B). The *bla*_DIM_ gene in pBJ86 was located upstream of the transposable element Tn*3* and in a Class 1 integron, with the gene arrangement *intI*1−*bla*_DIM−1_-$aac\left(6^{\prime }\right)-lb-bla_{\text{OXA}-4}$*-aadA1*-Δ3′-CS. The truncation of 74 bp 3′-CS is likely due to the IS*30* family transposase IS*Pa59* insertion. The genetic environment downstream of *bla*_DIM_ is relatively conservative, while upstream there are many other ARGs inserted, such as the chloramphenicol resistance gene *catB* in pNY11173-DIM and p12969-DIM (Figure 2B). *QnrVC6* mediates quinolone resistance through targeted protection. Highly similar to p12969-DIM, the *qnrVC6* gene was located upstream of *intI1* and in a complex Class 1integron, with the gene arrangement *intI1-qnrVC6-aacA3-IS*110*-catB-qacE*Δ*1-sul1-IS*CR1*-floR-sul1-qacE*Δ*1*. The resistance gene upstream of *qnrVC6* has a duplicate copy of the gene arrangement *sul1-qacE*Δ*1-catB-IS*110*-aac(6*′*)-la*, with insertion sequences *IS*110 and *IS*CR1 on both sides. This phenomenon may be caused by inserting sequence events. Through BLASTn, we also found that the sequence composed of *qnrVC6* and its nearby upstream and downstream genes is highly similar to a segment on the AM23 chromosome of *Pseudomonas* sp. (Sequence ID: CP113432.1), indicating that this sequence may have undergone exchanges between chromosomes and plasmids (Figure 2C).

The MRR not only carries multiple ARGs but contains two similar operons responsible for mercury resistance and their regulator, *merRTPADE*. Through BLASTn, it can be found that there are highly similar regions on both plasmids and chromosomes of *Pseudomonas* sp. to the region containing mercury resistant genes on pBJ86, and their similarity can reach 100%. For example, a fragment on the chromosome of *Pseudomonas* sp. 2022CK-00068 (sequence ID: CP124658.1) and *Pseudomonas* sp. BAMCP07-48 (sequence ID: CPU15377.1), and a segment on the plasmid of *Pseudomonas* sp. AR_0353 (sequence ID: CP027173.1). Additionally, *Tn21* can always be found on the flank of the mercury resistance gene (Figure 2D).

### 3.4 Conjugative system on plasmids

**Figure 3:**
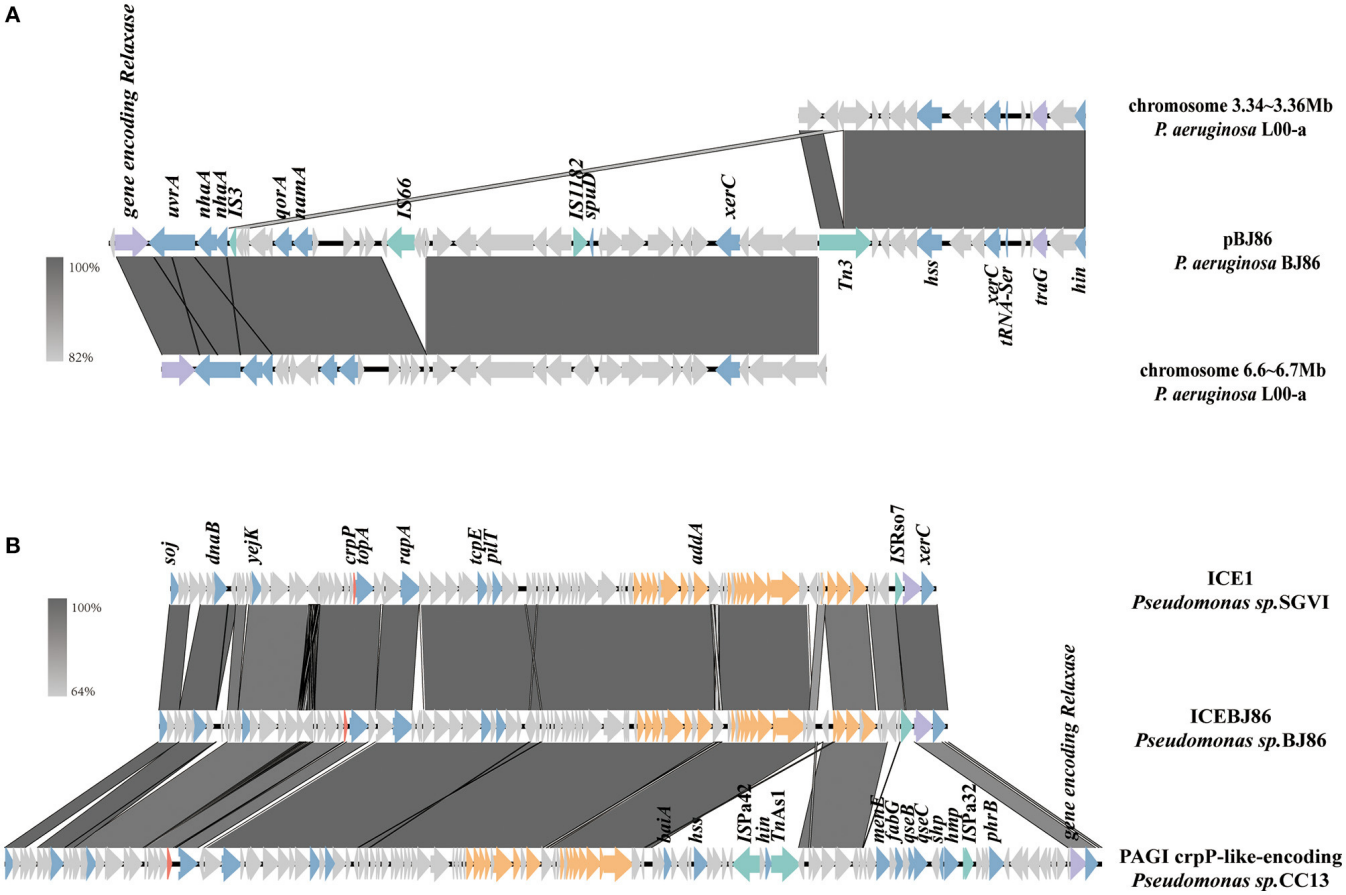
Schematic presentation of conjugative systems. Genes are presented as arrows, with the arrowhead indicating the direction of transcription. Genes involved in antimicrobial and heavy metal resistance are in red, mobile elements are in green, genes involved in the Type 4 secretion system are in orange, genes encoding relaxase and T4CP are in purple, genes involved in other functions are in blue, and genes involved in undetermined coding function are in light gray. Homologous segments generated by a BLASTn comparison (>64% identity) are shown in gray boxes. The alignment of genetic contexts of the conjugative system in plasmid pBJ86 **(A)** and ICEBJ86 **(B)** on the chromosome of BJ86 are with similar sequences.

At the position of plasmid pBJ86 420 to 500 kb, a variable region (VR) with a length of ~80 kb exhibits low alignment with its similar plasmids. This region does not contain any resistance genes, but genes encoding relaxase and type IV coupling protein (T4CP) can be found in this region, which is closely related to the mobility of the plasmid. The sequence containing both the genes encoding Relaxase and T4CP (*traG*) were highly similar to the sequence on the chromosome of *P.aeruginosa* L00-a (Sequence ID: CP097383.1), with 100% identity at 95% coverage (Figure 3A). In contrast with the chromosome of *P.aeruginosa* L00-a, there are some mobile elements on this sequence of pBJ86, like *IS*66, *IS*1182, and *Tn*3, which may mediate the insertion of genes encoding the conjugative system.

### 3.5 Genetic contexts of ICE*BJ86* on chromosome

On the chromosome of BJ86, there is an integrative and conjugative element (ICE), ICE*BJ86*, which carries a fluoroquinolone resistance gene *crpP* and a gene region encoding type IV secretion system (T4SS). The GC content of ICEs is usually lower than their host strains. The typical insertion sites are tRNA or other highly-conserved genes (Sun et al., 2023). In this study, the GC content of ICE*BJ*86 is 60.45%, 5.44% less than that of chromosome and inserted into the tRNA^Lys^ locus. Additionally, there are 54 bp direct repeats on both sides of it. Comparing the sequence of ICE*BJ*86 to those of NCBI, it was found that multiple strains of *Pseudomonas sp*. all carried this mobile element, such as *Pseudomonas sp*. SGVI (GenBank: KT887560.1) and *Pseudomonas sp*. 2021CK-01197 (GenBank: CP124643.1) (Figure 3B). However, *Pseudomonas* sp. SGVI was isolated from France in 1992 as ST111 type, and *Pseudomonas* sp. 2021CK-01197 from the United States in 2021 as ST308 type, with significant differences in time, location, and ST type, indicating that this type of ICE tends to be inherited through horizontal rather than vertical transmission.

### 3.6 Conjugation transfer experiment

Screening for resistance genes and MLST typing through second-generation sequencing data susceptibility testing confirmed that the plasmid pBJ86 could be transferred to rifampicin-resistant *Pseudomonas aeruginosa* PAO1, with frequencies of 6.24 × 10^−7^ transconjugants per donor cell. Firstly, the MLST typing of the donor strain BJ86 is ST2446, while the MLST typing of the recipient strain PAO1 is consistent with that of the transconjugant T86, both ST549. Secondly, based on the screening results of resistance genes, all 13 resistance genes carried by pBJ86 can also be screened on the transconjugant T86. However, we did not identify the fluoroquinolone resistance gene *crpP* carried by ICE*BJ86* on the chromosome of BJ86 on T86. Lastly, the sensitivity test further confirmed the success of the conjugation transfer experiment. As shown in Table 2, in comparison with PAO1, the transconjugant T86 showed increased MICs of cefepime, ceftazidime, ciprofloxacin, gentamicin, imipenem, levofloxacin, meropenem, norfloxacin, piperacillin-tazobactam, and tobramycin. Among them, the antibiotic sensitivity level of cefepime, ceftazidime, gentamicin, meropenem, and piperacillin-tazobactam has changed from sensitivity to resistance. The antibiotic sensitivity level of imipenem and norfloxacin has changed from intermediate to resistant. The antibiotic sensitivity level of tobramycin has changed from sensitivity to intermediate.

**Table 2:** Comparison of antimicrobial susceptibility test results before and after conjugation transfer experiment.

**Strains**	**MIC (mg/L)**														
	**AN**	**ATM**	**FEP**	**CAZ**	**CIP**	**CL**	**GM**	**IMP**	**LVX**	**MEM**	**NOR**	**TZP**	**TM**	**FOS**	**TGC**
BJ86	≤ 8(S)	8(S)	>16(R)	>32(R)	>4(R)	≤ 1(S)	>8(R)	>8(R)	>8(R)	>8(R)	>8(R)	>64/4(R)	>8(R)	64^a^	8
PAO1	≤ 8(S)	≤ 2(S)	≤ 1(S)	2(S)	2(R)	≤ 1(S)	≤ 2(S)	4(I)	4(R)	1(S)	8(I)	≤ 4/4(S)	≤ 2(S)	128	4
T86	≤ 8(S)	≤ 2(S)	>16(R)	>32(R)	>4(R)	≤ 1(S)	>8(R)	>8(R)	>8(R)	>8(R)	>8(R)	>64/4(R)	8(I)	64	8

AN, Amikacin; ATM, Aztreonam; FEP, Cefepime; CAZ, Ceftazidime; CIP, Ciprofloxacin; CL, Colistin; GM, Gentamicin; IMP, Imipenem; LVX, Levofloxacin; MEM, Meropenem; NOR, Norfloxacin; TZP, Piperacillin-Tazobactam; TM, Tobramycin; FOS, Fosfomycin w/G6P; TGC, Tigecycline.

S, susceptible; R, resistance; I, intermediate.

^a^The sensitivity of the donor bacteria BJ86, recipient bacteria PAO1, and transconjugant T86 to fosfomycin is lower than the ECOFF value (ECOFF ≤ 128 mg/L).

**Figure 4:**
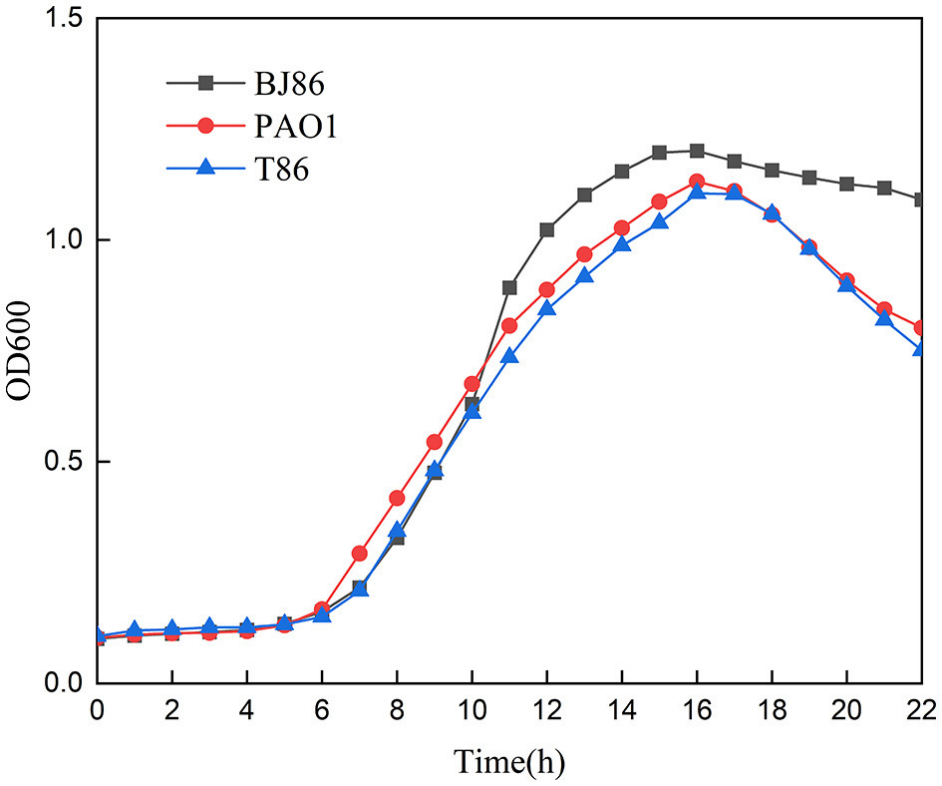
Growth curve of the donor stain BJ86, the recipient stain PAO1, and the transconjugant T86.

In addition, the growth curve of the receptor strain PAO1 before and after conjugation showed that there is no significant difference in the lag phase and logarithmic phase, and the OD value of PAO1 in the logarithmic phase is slightly higher than T86 (Figure 4). However, compared to the donor strain BJ86, which has a longer stationary phase, they both rapidly enter the decline phase after a brief stationary phase (about 1 h).

## 4 Discussion

As of 2022, the largest plasmid found to be carried by *P. aeruginosa* is a multidrug resistance plasmid pPAG5, which was found in a clinical strain of *P. aeruginosa* PAG5 isolated from a patient's urine, with a size of 513.3 kb (Li et al., 2022). Compared to pPAG5, pBJ86 has a larger size of 522.5 kb, and they both belong to megaplasmids. The emergence of megaplasmids is related to coping with different selection pressures, as they typically carry more resistance genes to enhance the adaptability of host microorganisms or (and) more effectively maintain their stability and transmission. In the *Pseudomonas*, these megaplasmids have the characteristics of high stability, low adaptation cost, and efficient transmission, making them effective vehicles for gene exchange. In *Salmonella Enterica, Agrobacterium tumefaciens*, and *Acinetobacter*, the megaplasmids carried by them also play an important role in the spread of AMR and tumor formation (Gordon and Christie, 2014; Cazares et al., 2020; Hall et al., 2022).

Plasmid pBJ86 carries many resistance-related genes, such as the genes encoding an efflux pump *tmexCD-oprJ*, which can mediate multiple antibiotic resistance, and the repressor *nfxB*, which mainly regulates its expression. Several mutations in *nfxB* have been related to the increased expression of *tmexCD-oprJ*, such as nucleotide deletions and missense and non-sense mutations. When overexpressed, it is associated with resistance to several antibiotics. In addition, recent studies have shown that *tmexCD-oprJ* is directly associated with tigecycline resistance and reduced sensitivity to other antibiotics under laboratory conditions (Lv et al., 2020; Lorusso et al., 2022). There is a significant truncation of *nfxB* on the plasmid pBJ86, so we tested the MIC of tigecycline against donor bacteria, recipient bacteria, and transconjugant by an antimicrobial susceptibility test *in vitro*. The results showed that the resistance to tigecycline increased from 4 to 8 μg/mL after conjugation. Compared with tetracycline, tigecycline has a broader antimicrobial spectrum and more potent antibacterial activity and can overcome the generation of a tetracycline resistance mechanism in most bacteria. Tigecycline has good antibacterial activity against most Gram-positive and Gram-negative aerobic and anaerobic bacteria *in vitro*, including *S. aureus, Enterococcus* spp*., S. pneumoniae, Haemophilus influenzae, Enterobacteriaceae*, and *Bacteroides* spp. (Pankey, 2005). For multidrug-resistant Gram-negative bacteria, especially carbapenem-resistant *Enterobacteriaceae*, tigecycline is a last line of defense drug together with polymyxin. *Pseudomonas aeruginosa* is naturally resistant to tigecycline (Dean et al., 2003), and tigecycline is usually not included in the clinical treatment of *Pseudomonas aeruginosa* infection (Pulmonary Infection Assembly of Chinese Thoracic Society, 2022). A *tmexCD-oprJ-*like gene cluster has been identified on the plasmids of several *Pseudomonas sp*. strains in the Genbank sequence (Bassetti et al., 2018a). If *tmexCD-oprJ* can become widespread among different species strains through plasmids and other mobile elements and mediate tigecycline resistance of bacteria coexisting with it, then we may need to re-examine the public health significance of the *tmexCD-oprJ* carried by a plasmid of *Pseudomonas* sp. In addition, pBJ86 also carries ARGs that mediate multiple antibiotic resistance, such as *bla*_DIM−1_, *bla*_OXA − 4_, *qnrVC6*, and *aac(6*′*)-la*, which mediate β-lactams, quinolone, and aminoglycoside antibiotic resistance. Although the *bla*_DIM_ gene has been detected worldwide (Leski et al., 2013; Sun et al., 2016; Tran et al., 2021; Delgado-Blas et al., 2022), compared to other commonly detected MBL genes such as *bla*_IMP_, *bla*_VIM_, and *bla*_NDM_„ pathogenic microorganisms carrying the *bla*_DIM_ gene are still less reported. Many studies have not included the *bla*_DIM_ gene in screening strains carrying the MBL gene using Polymerase Chain Reaction (PCR) technology (Murugan et al., 2018; Chen et al., 2022; Delgado-Blas et al., 2022). However, *bla*_DIM−1_, like other MBL genes, can also hydrolyze almost all β-lactams, which seriously hinders the effectiveness of clinical treatment (Tada et al., 2017). We should strengthen the monitoring of this MBL gene in future research.

In addition to possessing many ARGs, pBJ86 carries two structurally similar HRGs, *merRTPADE*. These two MRGs are located in the MRR region of the plasmid pBJ86, adjacent to the aminoglycoside resistance genes. Elements carrying HRGs are often detected in contaminated environments, and sublethal concentrations of heavy metals can promote the horizontal transfer of plasmids carrying ARGs (Zhang et al., 2018). Unlike antibiotics, metals are not easily degraded, so that they can bring sustained and stable selection pressure to microorganisms. Some pathogenic strains of *Pseudomonas* have already established environmental hosts, and resistance genes can be horizontally transferred from environmental organisms to human symbionts, which can bring direct public health impacts (Baker-Austin et al., 2006). In recent years, more and more studies have detected heavy metal resistance genes in clinical isolates, which typically coexist with various ARGs on mobile elements (Perez-Palacios et al., 2021; Li et al., 2022), and may be related to the co-selection mechanism between resistance genes. The co-selection mechanism includes co-resistance and cross-resistance. They all lead to the same consequence: the development of resistance to one antibacterial agent accompanied by resistance to another agent (Baker-Austin et al., 2006). Wireman et al. (1997) demonstrated that strains with the *mer* genes are more likely to develop multiple drug resistance than strains without the *mer* genes. Other studies have shown that *mer* operons that can co-transfer genes related to β-lactams, aminoglycosides, quinolones, sulfonamides, trimethoprim antibiotics, and disinfectants are of great significance for the transmission of several determinants of antimicrobial resistance (Perez-Palacios et al., 2021). Their studies have again demonstrated the correlation between metal and antibiotic resistance. In the future, we can continue to explore the mechanisms by which metal pollutants enhance the occurrence and transmission of antibiotic resistance in pathogenic microorganisms.

Compared to other plasmids carrying the genes *tmexCD-oprJ*, *bla*_DIM_, and *qnrVC6*, such as the plasmids containing DIM, including the first one discovered in *Pseudomonas stutzeri* in 2007 and one found to carry the DIM-1 variant DIM-2 in *Pseudomonas putida* in 2013, these plasmids have not been successfully transferred to the recipient strains. Similarly, the IncpRBL16 plasmid carrying *tmexCD3-toprJ3* found in *P. aeruginosa* could not conjugate transfer successfully (Dong et al., 2022). The plasmid pBM413 carrying *qnrVC*6 was preliminarily determined immobile due to its lack of genes encoding relaxase and T4SS. To our knowledge, pBJ86 is the first plasmid to carry *tmexCD-oprJ*, *bla*_DIM_, and *qnrVC6* simultaneously that can be successfully conjugated.

Compared to the other three methods of HGT [natural transformation, transduction, and vesicle-mediated transfer (Jiang et al., 2022)], conjugation is the most common mechanism of HGT and that with the broadest host range (Amábile-Cuevas and Chicurel, 1992). Conjugative systems involve an origin of transfer (oriT), a relaxase, a type IV coupling protein (T4CP), and a type IV secretion system (T4SS) (Getino and De La Cruz, 2018). According to the ability and characteristics of horizontal transfer, plasmids can be divided into three categories: conjugative plasmids, mobilizable plasmids, and non-conjugative plasmid. Among them, mobilizable plasmids only contain DNA transfer and replication systems but do not contain mating pair formation (Mpf). That is, they only contain Relaxase and T4CP. This type of plasmid often requires channels encoded by Mpf of another genetic element present in the cell for conjugation transfer, and Mpf is a form of T4SS (Smillie et al., 2010). Through gene level analysis, we can screen genes encoding T4CP and Relaxase in the low alignment region of plasmid pBJ86, but no genes encoding T4SS were found. Therefore, pBJ86 belongs to the mobilizable plasmid. Due to the successful conjugation transfer of pBJ86 and the absence of other conjugative plasmids in *Pseudomonas aeruginosa* BJ86, we subsequently analyzed the chromosomes of BJ86. The analysis results show that there are two T4SSs on the chromosome of BJ86, one located in ICE and the other in the chromosome genome outside of ICE. Further experimental research is needed to determine which T4SS-mediated plasmid conjugation is involved. Comparison of growth curves of receptor strain PAO1 before and after conjugation to the plasmid pBJ86 indicates that pBJ86 did not increase fitness costs or gains to the host bacteria. The rapid decline of T86 and PAO1 after the logarithmic phase may be a characteristic of the receptor strain PAO1 itself. Besides that, the toxin-antitoxin (TA) systems on plasmids can perform post segregational killing (PSK), thereby ensuring plasmid stability by killing plasmid-free daughter cells. However, we did not annotate the known TA systems on the plasmid pBJ86, indicating that strains carrying plasmid pBJ86 are likely to lose their plasmids after multiple subcultures (Hernández-Ramírez et al., 2017).

Like plasmids, ICE conjugation transfer also requires a complete system. There is an ICE*BJ86* with a complete conjugation system on the chromosome of BJ86. However, WGS analysis of the transconjugant T86 shows that its genome does not contain the ARG *crpP* carried by ICE*BJ86*, which means that ICE*BJ86* did not enter the receptor bacteria through conjugation, which may be because ICEs are quiescent elements located on the host chromosome in most cases, as activating ICEs can bring additional stress to the host strain. Only under certain conditions can ICEs be induced, such as SOS response cell-cell signaling (quorum sensing), a selective advantage that the ICE provides to the host (Johnson and Grossman, 2015).

The selective pressure of the environment is forcing the evolution rate of microorganisms to accelerate day by day, and resistance mechanisms that can render multiple drugs ineffective have emerged and spread at an astonishing rate. This is a source of concern and corresponding strategies must be devised. In recent years, more and more research and reviews have focused on the factors that affect the process of conjugative transmission, and we find that many endogenous and exogenous factors have an impact on the complex process. From the perspective of endogenous factors, the characteristics of bacteria themselves, such as host bacterial pilus morphology, restriction modification (RM), and CRISPR Cas systems, as well as the exclusion and susceptibility inhibition of plasmids, are common factors affecting the conjugation process (Getino and De La Cruz, 2018). From the perspective of exogenous factors, antibiotics [ampicillin, gentamicin, and tetracycline (Wang et al., 2023)], non-antibiotic pharmaceuticals [antipyretic analgesics acetaminophen, antiepileptic drug carbamazepine, and anticancer drug paclitaxel (Yang et al., 2022)], chemicals [copper ions and other ionic liquids (Wang et al., 2015; Song et al., 2021)], pollutants generated by human activities in the external environment [microplastics and disinfection by-products (DBPs) (He et al., 2022; Weise et al., 2022)], and even substances once thought to be able to sterilize and inhibit bacteria in the external environment, will also accelerate the spread of ARGs. Indeed, current research suggests that many substances can inhibit this process. Conjugation inhibitors (COINs) have been used to target specific components of conjugation systems to inhibit the process of conjugation transfer, such as blocking the activity of relaxase in receptor bacteria by using relaxase inhibitors, inhibiting the formation of conjugation transfer channels by affecting Mpf, and preventing contact between donor bacteria and receptor bacteria by interfering with the function of conjugative pilus (Getino and De La Cruz, 2018). Synthetic fatty acids and MoS2-decorated nanocomposite Fe2O3@MoS2 are among the current strategies (Getino et al., 2015; Wang et al., 2019).

## 5 Conclusion

In conclusion, this study identified and characterized a 522.5 kb mobilizable megaplasmid, pBJ86, from a clinical isolate *P. aeruginosa* strain, BJ86. To our knowledge, this is the largest plasmid found so far in *P. aeruginosa*. Plasmid pBJ86 carries multiple resistance genes, like the antibiotic resistance-related genes *tmexCD-oprJ*, *bla*_DIM−1_, and *qnrVC6*, and the mer operon system with resistance to mercury. We analyzed the genetic context of the abovementioned genes and the related genes involved in pBJ86 conjugation transfer. So far as we know, pBJ86 is the first plasmid to carry *tmexCD-oprJ*, *bla*_DIM−1_, *qnrVC6*, and the *mer* operon system simultaneously and that can be successfully conjugated. The emergence and transfer of the abovementioned ARGs pose a severe threat to the effectiveness of clinical treatment. We should adopt a more systematic approach to monitor mobile elements carrying ARGs and take adequate measures to curb the horizontal spread of ARGs. This research may provide discoveries and insights into the genomic diversity and molecular evolution of *P. aeruginosa*., which helps to understand the emergence of multidrug-resistant bacteria and the transmission mechanism of resistance genes.

## Data availability statement

The datasets presented in this study can be found in online repositories. The names of the repository/repositories and accession number(s) can be found in the article/Supplementary material.

## Ethics statement

Ethical approval was not required for the studies on humans in accordance with the local legislation and institutional requirements because only commercially available established cell lines were used.

## Author contributions

LM: Conceptualization, Data curation, Formal analysis, Investigation, Methodology, Software, Validation, Visualization, Writing—original draft. YS: Conceptualization, Investigation, Methodology, Supervision, Writing—review & editing. DL: Methodology, Supervision, Writing—review & editing. YL: Methodology, Supervision, Writing—review & editing. LL: Methodology, Visualization, Writing—review & editing. KY: Writing—review & editing, Methodology, Validation. MJ: Project administration, Writing—review & editing, Funding acquisition. DW: Project administration, Resources, Writing—review & editing, Conceptualization, Methodology. QW: Formal analysis, Funding acquisition, Project administration, Resources, Supervision, Writing—review & editing.
